# Bullöse Arzneimittelreaktion nach Gabe von Pembrolizumab – 2 Fallberichte

**DOI:** 10.1007/s00105-022-05018-0

**Published:** 2022-06-09

**Authors:** L. Golle, C. Michl, B. Kreft

**Affiliations:** Universitätsklinik und Poliklinik für Dermatologie und Venerologie, Universitätsklinikum Halle (Saale), Martin-Luther-Universität Halle-Wittenberg, Ernst-Grube-Str. 40, 06120 Halle (Saale), Deutschland

**Keywords:** Toxisch epidermale Nekrolyse, Checkpointinhibitor, Pembrolizumab, Bullöse Arzneimittelreaktion, Histologie, Toxic epidermal necrolysis, Checkpoint-inhibitor, Pembrolizumab, Bullous drug reaction, Histology

## Abstract

Zu den schweren, blasenbildenden Arzneimittelreaktionen an der Haut gehören das Stevens-Johnson-Syndrom (SJS) und die toxisch epidermale Nekrolyse (TEN). Allopurinol, Antikonvulsiva, Sulfonamidantibiotika und nichtsteroidale Antirheumatika vom Oxicam-Typ sind wiederholt als Auslöser beschrieben. Zunehmend rücken auch Immuntherapien als Auslöser schwerer Hautreaktionen in den Fokus. Vorgestellt werden 2 Patienten mit bullösen Hauterscheinungen nach Gabe des Checkpointinhibitors Pembrolizumab. Da das klinische Bild nicht immer eine zweifelsfreie Einordnung zulässt, ist eine histologische Mitbeurteilung vielfach unverzichtbar.

## Abstract (en)

Severe, blistering, adverse drug reactions involving the skin include Stevens-Johnson syndrome (SJS) and toxic epidermal necrolysis (TEN). Allopurinol, anticonvulsants, sulphonamide antibiotics and non-steroidal anti-inflammatory drugs in the oxicam class have been repeatedly described as triggers. Increasingly, immunotherapies are also coming into focus as triggers of severe skin reactions. Two patients with bullous skin symptoms after administration of the checkpoint inhibitor pembrolizumab are presented. As the clinical picture does not always allow an unequivocal classification, a histological assessment is often indispensable.

## Falldarstellung 1

### Anamnese

Ein 67-jähriger Patient stellte sich mit seit 2 Tagen bestehenden, teils pruriginösen, berührungsempfindlichen, entzündlichen Hauterscheinungen am gesamten Integument in unserer Klinik vor. Zwei Monate zuvor war ein hepatisch und ossär metastasiertes Hypopharynxkarzinom mit positivem PD-L1-Rezeptorstatus erstdiagnostiziert worden. Der Patient wurde daraufhin einer Immuntherapie mit dem PD-1-Antagonisten Pembrolizumab (200 mg intravenös alle 3 Wochen) zugeführt. Zehn Tage nach dem 2. Zyklus waren dem Patienten erste Hauterscheinungen in Form stammbetonter Erytheme aufgefallen, die sich seitdem deutlich progredient zeigten. Die Dauermedikation (Candesartan, Dutasterid/Tamsulosin, Metoprolol, Pantoprazol, Torasemid) war in den vergangenen Monaten konstant. Lediglich Dexamethason 4 mg/Tag war 4 Wochen zuvor neu verordnet worden.

### Klinischer Befund

**Figure 1:**
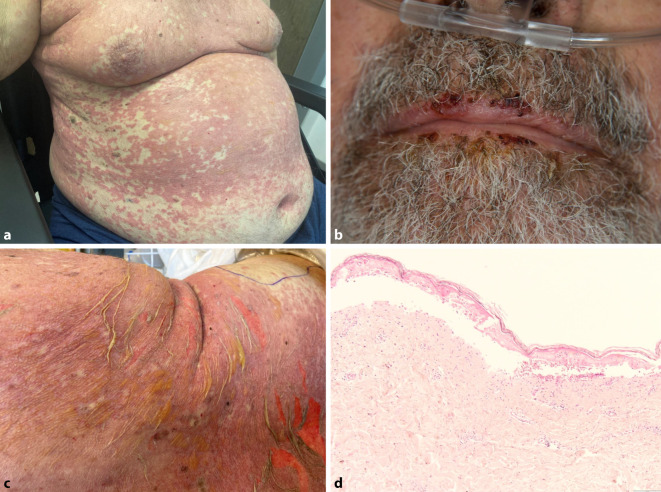

Wir sahen einen Patienten in deutlich reduziertem Allgemeinzustand mit einem vorwiegend stammbetonten, kleinfleckigen, makulopapulösen, teils urtikariell anmutenden Exanthem (Abb. 1a). An den Fußrücken beidseits zeigten sich schlaffe, nichtverschiebliche Blasen, betont an Grenzflächen von mechanisch beanspruchten Arealen. Direktes und indirektes Nikolski-Zeichen waren negativ. Auffällig waren eine flache Erosion an der Glans penis und ein ausgeprägter Sklerenikterus ohne Zeichen einer Konjunktivitis. Die Lippen erschienen trocken mit einzelnen hämorrhagischen Krusten (Abb. 1b). Enoral zeigten sich Candida-assoziierte weiße, abstreifbare Beläge ohne erosive Mundschleimhautveränderungen. Eindrücklich imponierte eine progrediente stammbetonte Blasenbildung an der Haut innerhalb von 2 h (Abb. 1c).

### Labor

C‑reaktives Protein 59,5 mg/l (Normalwert < 5 mg/l), Leukozyten 14,77 Gpt/l (Referenzbereich 3,90–10,40 Gpt/l), Procalcitonin 0,52 µg/l (Normalwert < 0,50 µg/l).

### Histologie

Histologisch zeigte sich eine subepidermal vom Korium abgelöste, orthokeratotisch verhornte nekrotische Epidermis. Im Stratum papillare fanden sich schüttere perivaskuläre und interstitielle lymphozytäre Infiltrate mit neutrophilen Granulozyten entsprechend dem Bild einer toxisch epidermalen Nekrolyse (Abb. 1d).

### Diagnose

Toxisch epidermale Nekrolyse (TEN) auf Pembrolizumab.

### Therapie und Verlauf

Der Patient wurde auf einer Metalline-Folie (Firma: Lohmann&Rauscher) gelagert und erhielt eine intravenöse Therapie mit Methylprednisolon 1,5 mg/kg Körpergewicht pro Tag über 4 Tage. Zudem erfolgte eine supportive Intensivbehandlung. Die Lokaltherapie der erosiven Areale erfolgte mit wirkstofffreier Paraffingaze und antiseptischen Gelzubereitungen bei atraumatischen Verbandswechseln unter sterilen Kautelen. Wir veranlassten eine interdisziplinäre Mitbetreuung des Patienten.

Nach zunächst erfreulicher Stabilisierung des Hautbefundes kam es im Verlauf jedoch zu einer progredienten Exsikkose mit Tachykardie, produktivem Husten und deutlicher Verschlechterung der Lungenfunktion, sodass eine kalkulierte intravenöse Antibiotikatherapie mit Meropenem und Amikacin indiziert war. Bei Auftreten eines Lungenödems mit progredientem Sauerstoffbedarf musste der Patient im weiteren Verlauf intubiert und invasiv beatmet werden. Komplikativ trat eine Laktatazidose hinzu, welche die Einleitung einer kontinuierlichen Dialysebehandlung erforderlich machte. Trotz aller geschilderter intensivmedizinischer Maßnahmen wurde der Patient zunehmend katecholaminpflichtig und verstarb am 6. stationären Behandlungstag.

## Falldarstellung 2

### Anamnese

Die Aufnahme des 64-jährigen Patienten erfolgte aufgrund eines seit etwa einer Woche bestehenden, generalisierten, pruriginösen und teils schmerzhaften Exanthems mit Blasenbildung. Der Patient hatte aufgrund eines zerebral metastasierten, nichtkleinzelligen Lungenkarzinoms (NSCLC) Pembrolizumab 200 mg mit Carboplatin 330,4 mg und nab-Paclitaxel 418 mg einmalig 3 Wochen zuvor erhalten. Ebenso hatte er Cotrimoxazol aufgrund einer Bakteriämie nach Spondylodese bis eine Woche vor Aufnahme erhalten. Eine intravenöse Therapie mit Prednisolon 100 mg und Dimetindenmaleat 4 mg war zum Aufnahmezeitpunkt bereits einmalig verabreicht worden.

### Klinischer Befund

**Figure 2:**
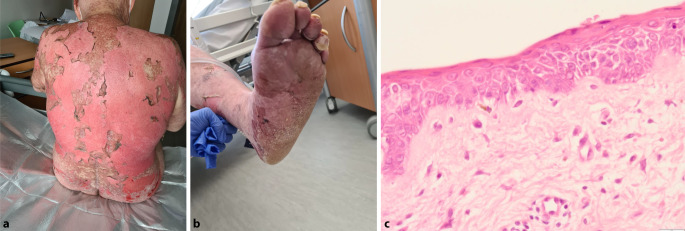

Zum Aufnahmezeitpunkt imponierte ein makulopapulöses, konfluierendes, stammbetontes Exanthem unter Betonung des Rückens mit großflächigen Erosionen. Weniger betroffen waren die proximalen Extremitätenabschnitte und Fußsohlen beidseits, zudem geringgradige Erosionen an der Nasenspitze (Abb. 2a,b). Es bestand kein Anhalt für eine Schleimhautbeteiligung.

### Labor

C‑reaktives Protein 130 mg/l (Normalwert < 5 mg/l), normwertige Leukozyten, Procalcitonin im Referenzbereich.

#### Serologie

Mykoplasmen‑, Hepatitis-A–C- und HIV-Serologie negativ.

### Röntgenaufnahme des Thorax

Ausgedehnte Verschattung im Bereich des linken Ober- und Mittelfeldes vereinbar mit bekanntem NSCLC.

### Histologie

Histologisch zeigte die Stanzbiopsie einer nichtbullösen Läsion eine schmale Parakeratose mit epidermaler Dysmaturation und fokaler Spongiose, sägezahnartigen Reteleisten sowie einzelnen apoptotischen Keratinozyten mit vakuolärer Degeneration und Pigmentinkontinenz im ödematösen Stratum papillare mit schütteren perivaskulären lymphozytären Infiltraten (Abb. 2c).

### Diagnose

In der Zusammenschau der klinischen und histologischen Befunde stellten wir die Diagnose eines bullösen lichenoiden Arzneimittelexanthems auf Pembrolizumab.

### Therapie und Verlauf

Wir führten zunächst die orale Prednisolontherapie mit 30 mg/Tag für 2 Tage fort. Supportiv erfolgte eine antiseptische, atraumatische Lokaltherapie mit Anwendung potenter Glukokortikosteroide sowie bilanzierter intravenöser Volumensubstitution und adaptierter Analgesie. Darunter bildeten sich die beschriebenen Hauterscheinungen innerhalb von 10 Tagen zurück.

## Diskussion

Kutane Nebenwirkungen von Checkpoint-Inhibitoren werden in der Literatur mit einer Häufigkeit von 8,7–37 % angegeben [1]. Bullöse Hautreaktionen mit Epidermolyse werden selten beobachtet [2–4]. Unter PD‑1 bzw. PD-L1-Therapie sind auch bullöse lichenoide Arzneimittelexantheme [5], Fälle von therapieinduziertem bullösem Pemphigoid [6] oder aber schwere, potenziell lebensbedrohliche Hautreaktionen wie SJS oder TEN [3, 4] beschrieben.

Über schwere unerwünschte kutane Arzneimittelreaktionen auf den PD-1-Antagonisten Pembrolizumab wurde seit Markteinführung wiederholt berichtet [5, 7]. Der Wirkstoff ist bei der ersten hier vorgestellten Kasuistik als wahrscheinlichster Auslöser der TEN zu werten. Es wird derzeit angenommen, dass die Blockade der Bindung von PD‑1 und PD-L1 zu einem Verlust der T‑Zell-Homöostase in Haut und Schleimhäuten führt, was in einem Versagen der Immuntoleranz und somit selbstgesteuerten zytotoxischen Reaktionen resultiert [1, 4].

Histopathologisch wird eine Akkumulation von CD8+-T-Zellen an der dermoepidermalen Junktionszone ebenso wie eine CD8+-T-Zell-Exozytose in die Epidermis mit apoptotischen Keratinozyten beobachtet [4]. Dieser Mechanismus kann auch bei schweren immunvermittelten Hauterkrankungen wie bei akuter GVHD und SJS/TEN beobachtet werden. Genexpressionsanalysen aus Hautläsionen von mit Anti-PD‑1 behandelten Patienten ergaben ein Genexpressionsprofil, welches dem einer SJS/TEN ähnelt. Immunhistochemisch imponiert in betroffener Haut eine Hochregulierung proinflammatorischer Chemokine wie CXCL9, CXCL10 und CXCL11 sowie von zytotoxischen Mediatoren wie PRF1 (Perforin 1) und GZMB (Granzym B) ebenso wie dem proapoptotischen Molekül FASLG (FAS-Ligand) und von PD-L1 [4].

Auch in Fall 2 hat Pembrolizumab offenbar zu bullösen Hauterscheinungen mit klinisch allerdings blandem Verlauf, rascher Abheilungstendenz und gering ausgeprägter Schleimhautbeteiligung geführt. In der Abgrenzung zu Fall 1 liegt hier in Zusammenschau der klinischen und histologischen Befunde offenbar ein „bullöses lichenoides Arzneimittelexanthem“ vor. Ein möglicher Zusammenhang mit der Einnahme von Cotrimoxazol konnte nicht mit letzter Sicherheit ausgeschlossen werden, ebenso ist eine überlappende Toxizität mehrerer Arzneimittel grundsätzlich möglich [8].

Das klinische Bild des „bullösen lichenoiden Arzneimittelexanthems“ kann zunächst an eine TEN denken lassen, geht aber mit einer nur geringgradigen oder fehlenden Schleimhautbeteiligung einher [9]. Die histopathologischen Veränderungen zeigen sowohl bei lichenoiden Arzneimittelreaktionen als auch bei SJS/TEN nekrotische Keratinozyten mit subepidermaler Spaltbildung. Wegweisend für die lichenoide Arzneimittelreaktion sind die ausgeprägteren lymphozytären Infiltrate sowie, wie im hier geschilderten 2. Fall, eine irregulär akanthotisch verbreiterte Epidermis, häufig mit Parakeratose einhergehend [10, 11].

Auffällig war im Fall 1, dass trotz klinisch und histologischer Charakteristika einer TEN die Affektion der Schleimhäute, wie sie meist regelhaft beim Vollbild einer klassischen TEN durch Allopurinol, Antikonvulsiva oder antibakterieller Sulfonamide gesehen wird, nur sehr dezent ausgeprägt war. Ähnliche Beobachtungen sind bereits publiziert worden [9, 12], sodass postuliert werden kann, dass es sich in Abgrenzung zur klassischen TEN hier eher um ein „TEN-artiges“ Krankheitsbild handelt, wenngleich die klinischen und histologischen Charakteristika sehr ähnlich erscheinen. Eine im Gegensatz zur klassischen TEN mildere Schleimhautbeteiligung und eine raschere Reepithelisierungstendenz könnten klinische Marker für eine Immuntherapie-vermittelte TEN sein [9, 10, 12]. Somit ist im Vergleich zur klassischen TEN die bullöse „TEN-artige Arzneimittelreaktion“ als möglicherweise prognostisch günstiger einzuschätzen. Ob dies allgemein so angenommen werden kann, wird man anhand zukünftiger Beobachtungen bewerten müssen. Der letztendlich letale Verlauf unseres Patienten (Fall 1) trotz Fehlen einer relevanten Schleimhautbeteiligung ist in diesem Zusammenhang überraschend und am ehesten im Zusammenhang mit der zugrundeliegenden Tumorerkrankung, den übrigen Begleiterkrankungen sowie dem komplikativ eingetretenen Lungenödem mit Sepsis zu werten.

Bullöse lichenoide Arzneimittelreaktionen, wie im hier dargestellten 2. Fall beschrieben, sind jedenfalls grundsätzlich differenzialdiagnostisch von einer TEN oder einem „TEN-artigen Exanthem“ abzugrenzen, insbesondere da der Verlauf deutlich milder ist. Die bisweilen klinisch schwierige Einordnung der verschiedenen Krankheitsbilder kann durch ergänzende histologische Untersuchungen unterstützt werden (s. auch Tab. 1). Dabei ist differenzialdiagnostisch immer auch ein generalisiertes bullöses fixes Arzneimittelexanthem oder ein therapieinduziertes bullöses Pemphigoid in Betracht zu ziehen.

**Table 1:** 

	Patient 1	Patient 2
Alter (Jahre)	67	64
Geschlecht	M	M
*Zugrundeliegende Tumorerkrankung*	Hypopharynxkarzinom Stadium IV (ossär *metastasiert*)	Nichtkleinzelliges Lungenkarzinom Stadium IV (zerebral *metastasiert*)
Tumortherapie	Pembrolizumab 200 mg q 3 wk. Hauterscheinungen 10 Tage nach 2. Zyklus	Pembrolizumab 200 mg + Carboplatin 330,4 mg + nab-Paclitaxel 418 mg einmalig 3 Wochen vor Hauterscheinungen
Dermatologische Einschätzung bullöser Reaktion	Rasch progredientes, großflächiges, stammbetontes Erythem mit schlaffen Blasen und starker AZ-Minderung Weitestgehend fehlende Schleimhautbeteiligung **TEN auf Pembrolizumab**	Eher stagnierender bis langsam progredienter Hautbefund mit wenig entzündlicher Aktivität Keine Schleimhautbeteiligung **Bullöses lichenoides Arzneimittelexanthem auf Pembrolizumab**
Therapie	Intensivmedizinische Betreuung Non-adhäsive und antiseptische Lokalbehandlung Methylprednisolon 1,5 mg/kgKG/Tag i.v. über 4 Tage	Bilanzierte Volumensubstitution und Analgesie Topische Glukokortikosteroide sowie topisch antiseptische Lokalbehandlung Fortführung Prednisolon 30 mg/Tag p.o. für 2 Tage
Verlauf	Trotz intensivmedizinischer Therapie Versterben innerhalb von 6 Behandlungstagen	Vollständige Abheilung

Die hier aufgeführte tabellarische Gegenüberstellung der beiden Fälle ist als Ergänzung bereits publizierter tabellarischer Aufstellungen zu verstehen [2, 5, 9, 13]

Die klinische Abgrenzung der einzelnen Krankheitsentitäten ist insbesondere deshalb von Bedeutung, da die Entscheidung zu treffen ist, ob aufgrund der manifesten Hauterscheinungen das dauerhafte Absetzen des auslösenden Arzneimittels geboten ist oder ob nach Nutzen-Risiko-Abwägung die Fortführung bzw. die vorsichtige Reexposition – ggf. unter begleitender symptomatischer Therapie mit z. B. systemischen Glukokortikosteroiden – vertretbar ist. In der Tumortherapie ist diese Abwägung bei vielfach begrenzten oder fehlenden Therapiealternativen von entscheidender Bedeutung.

Erwähnenswert ist zudem, dass Pembrolizumab eine lange Halbwertszeit von 23 Tagen besitzt [2]. Dies könnte eine pharmakokinetische Erklärung für prolongierte Verläufe unerwünschter Arzneimittelreaktionen sein [2, 8].

## Fazit für die Praxis

- Mit zunehmender Etablierung neuer Therapien muss mit neuartigen unerwünschten Arzneimittelwirkungen der Haut gerechnet werden.
- Bei bullösen Hauterscheinungen unter Therapie mit PD-1-Antagonisten sind TEN-artige Reaktionen von anderen blasenbildenden Erkrankungen abzugrenzen. Zu diskutieren ist, ob im Zusammenhang mit dem Auftreten bullöser kutaner Hautreaktionen unter PD-1-Antagonisten wie Pembrolizumab aufgrund der vielfach nur gering ausgeprägten Schleimhautbeteiligung und somit für die betroffenen Patienten quoad vitam günstigeren Prognose nicht passender der Begriff „TEN-artiges Exanthem“ verwendet werden sollte.
- Über das Absetzen oder die Fortführung der für die Hauterscheinungen ursächlichen Tumortherapie muss im Einzelfall unter Berücksichtigung der zur Verfügung stehenden Therapiealternativen nach sorgfältiger Nutzen-Risiko-Abwägung in Kooperation mit dem behandelnden Onkologen entschieden werden.
